# Xenotransfusion of Blood from Dog to Cat: Should Canine Blood Be Our First Choice for Feline Transfusion in Emergency Situations?

**DOI:** 10.3390/vetsci9030106

**Published:** 2022-02-28

**Authors:** Jack-Yves Deschamps, Nour Abboud, Françoise A. Roux

**Affiliations:** Emergency and Critical Care Unit—Nutrition, PathoPhysiology and Pharmacology (NP3) Unit, Oniris, Nantes-Atlantic College of Veterinary Medicine, La Chantrerie, CS 40706, CEDEX 03, 44 307 Nantes, France; nour.abboud@oniris-nantes.fr (N.A.); francoise.roux@oniris-nantes.fr (F.A.R.)

**Keywords:** transfusion, xenotransfusion, dog-to-cat, emergency, critical care, cat, feline

## Abstract

Despite the ability to determine feline blood types, the transfusion of canine blood to cats is still practiced in some countries. Xenotransfusion is effective—even if its effects only last for a few days—and is not associated with serious adverse effects. It avoids the need for blood typing, and most importantly, it avoids the transmission of intraspecific infectious agents, notably the feline leukemia virus (FeLV). Transfusion with canine blood is easier, quicker and less costly than transfusion with feline blood; it is less disagreeable for the donor. In the light of these arguments, when feline blood collected according to current guidelines is not available, in particular when the donor is not confirmed to be negative for the FeLV provirus, the authors consider it to be judicious to use canine blood for feline transfusion in emergency situations; this practice is preferable to inaction and to the inoculation of an infectious agent. Allotransfusion remains preferable in non-emergency situations as a treatment of chronic compensated anaemiae or if an appropriate donor (negative for FeLV provirus) is available. However, 2–4 days after a xenotransfusion, if a clinical alteration and a significant decrease in haematocrit are observed, a transfusion with cat’s blood confirmed to be negative for FeLV provirus should be performed. Xenotransfusion should never be used twice.

## 1. Introduction

A small number of publications discuss the risks and benefits of xenotransfusion of canine blood to cats. Until 2013, 61 cases were described in 4 publications dating back to the 1960s [1,2,3,4]. For 46 years, from 1968 to 2014, no publication appeared; the practice was considered to be outdated. Only with Catherine Bovens’s review [5], which examined past successes, was xenotransfusion brought back from oblivion, and authors once again occasionally began to report a few cases: as of 2019, 17 have subsequently been described [6,7,8,9,10,11,12], in addition to two in vitro studies [13,14]. Only in 2020 did a large series consisting of 49 cases become more published [15].

In their conclusions, all the authors emphasize the benefits of xenotransfusion in the cases they describe but believe that the practice should remain a rare option. In Le Gal’s series [15], clinicians only performed xenotransfusions if they considered the cat likely to die within 6 h without blood, and in the absence of feline blood products. With this conclusion, the authors attempt to acknowledge the empirical pragmatism at the heart of the scientific literature, which is dominated by respect for the species barrier.

Current guidelines concerning feline transfusion recommend using feline blood [16,17,18,19]. There are, however, powerful arguments in favor of more widespread use of canine blood for feline transfusion.

## 2. Emergency Use

The use of a canine donor has often been justified by the urgent need for transfusion. Anaemic cats often present in a critical condition, and are in need of immediate transfusion. This is the case in anaemiae secondary to traumatic or post-surgical haemorrhage, haemolysis, anticoagulant rodenticide ingestion, or with decompensation of a previously tolerated chronic anaemia. Anaemic cats which are not decompensating are rarely transfused. Feline transfusion is thus often an emergency, so xenotransfusion should be considered.

## 3. Benefits

The justification for emergency treatments are based on their immediate effectiveness and lack of adverse effects. Xenotransfusion with canine blood fulfils these two criteria. Except for cats that died or were euthanized during xenotransfusion, most reports agree on rapid improvement (within hours) in the clinical situation of cats that receive canine blood, just as is seen with allotransfusion. Admittedly, authors tend to publish individual cases which turn out well, but the old case series and the large series from Le Gal et al. [15] support this generally favorable course. This improvement is correlated with an increase in haematocrit; in Le Gal’s series [15], median haematocrit before transfusion was 10%, and median haematocrit 12 h after xenotransfusion was 25%. Mortality rate is rarely mentioned, but when it is, it is attributed to the primary disease, never as a result of the xenotransfusion [8,15].

When xenotransfusion was still practiced in human patients, similar benefits were seen, despite the lack of asepsis and the rudimentary catheterization techniques available, and despite the lack of anticoagulants. In 1868 Pierre-Cyprien Oré (1828–1890), a French physiologist, reported on 154 transfusions with animal blood (lamb, sheep, calf) to humans in his book [20]. He counted 64 cures, 20 improvements, 43 unchanged cases, 1 questionable case and 26 deaths. He found only a single failure connected with the transfusion, in the course of which death occurred immediately. He later described a second similar case of his own in the second edition of his book, but at the same time he related the case of a 42-year-old man with anaemia who received three transfusions of human blood without improvement, but who recovered rapidly with a transfusion of lamb’s blood [21]. Even then, Oré stressed that animal blood had the advantage of limitless supply, which is always available, and that it eliminates the risk to a human donor.

The benefits of allotransfusion are expected to persist for about 30 days [22]. The benefits of xenotransfusion of canine blood to cats are of limited duration because of the rapid destruction of canine erythrocytes by the cat’s antibodies—4 to 7 days in most studies and only 2 days in Le Gal’s series [15]—but if another transfusion is indicated once the initial crisis has passed, based on the clinical picture and a falling haematocrit, an allotransfusion conforming to current recommendations may still be given. Recently published clinical cases do not always report the need for a second transfusion. In recent reports, only a single transfusion was needed in 36/56 cases (64%) [6,7,8,9,10,11,15,23]; a second transfusion, intraspecific this time, was needed in 20/56 cases (36%): in the second cat reported by Euler [9], in 5/9 cats in Klainbart’s series [8] and in 14/39 cats in Le Gal’s series [15]. Thus, despite rapid haemolysis of the canine blood, a second transfusion around the fourth day has only been necessary in one third of cases.

## 4. Lack of Severe Adverse Effects

No severe transfusion reaction has been reported up to the present time. Rare minor immediate secondary effects have been seen (hyperthermia, tachypnoea) [3,15], neither different from nor more frequent than those observed in allotransfusions, and not significantly different between cats with a compatible or incompatible cross-match [15]. In their series of xenotransfusions, Le Gal et al. [15] saw only six (12%) cases of inconsequential febrile reactions and no other acute transfusion reactions; no particular reaction was reported in the nine cats (18%) that received a feline blood transfusion of feline packed red blood cells before the xenotransfusion. By the 4th day, because of the rapid haemolysis of canine erythrocytes, hyperbilirubinemia, possibly accompanied by icterus [15,23], is sometimes reported without clinical consequence. Older cases, which often attempted a second xenotransfusion (20/22 cases in Hessler’s series [1]) demonstrate the absolute contraindication of a second transfusion with canine blood, which was almost always fatal within a few minutes or hours after that time. As with any procedure, especially one which does not fall within current guidelines, the owners’ consent must be obtained. Owners should be clearly informed that their cat has received dog blood so that they can report this fact to their veterinarian if another transfusion is needed. In Le Gal’s series [15], all owners contacted remembered that their cats had received a xenotransfusion.

## 5. Ease of Collection

Dogs are more docile than cats. Donor cats must often be sedated, even sometimes anaesthetized; cardiomyopathies are not rare in cats, and anaesthesia could be dangerous. Dogs are more co-operative and do not require anaesthesia, or even sedation, especially for the small volumes needed for cats. The size of their veins permits blood collection from the cephalic veins in large dogs with minimal restraint, while collection in cats must be from the jugular vein, which requires greater physical or chemical restraint.

## 6. Blood Volume Required

The typical volume of blood collected for feline transfusion is 30 to 80 mL (in Le Gal’s series [15], the median volume transfused was 14.6 mL/kg). It does not represent a significant loss for medium to large dogs: 60 mL of whole blood is only about 2% of a Labrador’s blood volume, while it represents 25–30% of a donor cat’s blood volume. Large volumes of blood are sometimes required for cats [24], and the needs for xenotransfusion may be met by a single dog, while allotransfusion would require multiple feline donors, which accordingly increases the precautions required. The use of large volumes of canine blood has however not been documented, and it is probable that the risks may be higher. In Le Gal’ series [15], the volume transfused was up to 28 mL/kg; such a volume of fresh whole blood cannot be drawn from a single cat, and thus requires access to a bank of feline blood, or to have recourse to an eventual xenotransfusion as an adjunct to allotransfusion. With the same arguments, due to their ability to donate large volumes of blood, cattle were considered as source of blood for goats [25].

## 7. Natural Antibodies

Most cats belong to one of two blood types: type A or type B [19]. Cats have natural antibodies to erythrocytes of the other blood types of their species: type A cats have anti-type-B antibodies, and type B cats have a great number of antibodies against type A [19]. An allotransfusion with incompatible blood causes, at a minimum, haemolysis (in the case of a type-B donor and a type-A recipient), or at the worst death within a few minutes (in the case of a type-A donor and a type-B recipient) [26]. With cats it is thus not possible, as is the case with dog-to-dog transfusions, to perform a single transfusion without verifying compatibility; a cat must therefore not be transfused without prior blood typing, and ideally cross-matching. Use of canine blood does not expose the feline recipient to these dangers.

Most authors agree on the absence of natural antibodies in cats against dog erythrocyte antigens (and vice versa) and thus confirm the value of canine blood. This belief is based on compatibility tests obtained prior to transfusion, and on the absence of observed anaphylactic reactions. However, three publications have demonstrated the existence of such antibodies [9,13,14]; in these studies none of the cats was entirely negative on either major or minor cross-match. The authors conclude that there is theoretically a high risk of moderate to severe reaction. This risk has never been observed in vivo in the 127 published cases. In Le Gal’s series [15], major cross-matches were incompatible in 20 of 29 cases (69%) and minor cross-matches were incompatible in 8 of 26 cases (31%), but no severe immediate reaction was observed, and incompatibilities between donors and recipients did not predict the development of a delayed haemolytic transfusion reaction.

Lacking the ability to determine the blood type of the feline recipient (in a critical emergency [10] or in the case of conflicting test results [9]), the risk of transfusion reaction with canine blood is much lower than that associated with the use of blood from a cat chosen at random. In the case of xenotransfusion with canine blood, a dog may be selected even if its blood group is unknown. In Le Gal’s series [15], 35 of 49 cats (71%) received dog erythrocyte antigen (DEA)-1-positive packed red blood cells and 14 (29%) received DEA-1-negative packed red blood cells.

## 8. Rarity of Blood Type B

The prevalence of feline blood types varies from country to country; on average 85% of cats belong to type A, and 15% to type B [19,27]. In a country like France where the prevalence of type B is 10% [27], if the recipient belongs to type B, theoretically, it would be necessary on average to test 10 random cats to find a compatible donor. A veterinarian who performs feline blood transfusions should have a list of (indoor-only) type B cats available to give blood; these cats should always be available or be regular donors to a blood bank.

A similar situation is found with wild species, captive or not. The lack of knowledge of their biology, and the impossibility of finding a donor of the same species, has led to the successful practice of xenotransfusions using blood from a closely-related domestic species [28,29]. Xenotransfusion of blood from domestic dogs can be considered for captive non-domesticated canids [30]. Xenotransfusion’s benefits and lack of adverse effects should be studied in domestic animals such as the ferret, in which collecting and storing blood is problematic [31,32].

## 9. Rarity of and Problems with Feline Blood Banks

There is no synthetic blood product on the market that can be used in cats; Oxyglobin^®^, a commercially prepared bovine hemoglobin solution, is currently not available.

Establishing a feline blood bank allows immediate access to blood from a cat of a known blood type, and of known status regarding the principal feline infectious agents, but this entails a great deal of work for a clinic. Some University centers and large practices do have lists of potential donors which are typed that can be called in an emergency, thus avoiding the post-emergency destruction of a canine transfusion. Keeping a list of typed cat donors who could be contacted is a valid alternative, but it is not always possible to bring in the donor within the deadlines imposed by an emergency; it remains an excellent option if further transfusions are necessary after a xenotransfusion.

In Le Gal’s series [15], the clinicians performed a xenotransfusion only in the absence of feline blood products; the cases were drawn from two major university hospitals in the United Kingdom, emphasizing the challenge of building a feline blood bank. It is understandable that smaller-scale organizations decide to forego blood banking for its rare need. If the veterinary clinic has a canine blood bank but feline blood is not available, the use of canine packed red blood cells is preferable to the use of canine whole blood; in Le Gal’s series [15], all cats received packed red blood cells.

Facilities possessing a canine blood bank do not necessarily have a feline blood bank: cats are less co-operative donors, and it is not as easy to obtain quality donations with cats as with dogs. In fact, the collection bags currently available are not appropriate for feline blood collection. The 250 mL collection bags designed for human blood that are used for dogs have a needle that is very large for cats, and there is significant risk for jugular vein damage. It is possible to use a semi-closed system to improve safety [33]. The bags designed for cats have a smaller needle, but since they do not contain anticoagulants, it is necessary to withdraw citrate phosphate dextrose acid (CPDA) from a human bag, then to collect the donor cat’s blood with a butterfly catheter, then to inject it back into another bag [16,19,34]. Since the collection system is not closed, the risk of contamination during handling is increased. Aspiration and injection alter blood quality [35]. In a study carried out in ferrets, in which collection techniques are comparable, it was shown that the blood should be used within 7 days of collection [31]. To be useful, a blood bank must have enough blood, which implies a stock greater than estimated requirements. A certain amount of blood products will inevitably be discarded. Collection requires a great deal of work and expense, and donors are subjected to unjustified trauma.

## 10. Infection Risks

### 10.1. Conventional Infection Risks

The risk of retrovirus transmission is a major concern with blood transfusions. The case of human blood contaminated with the AIDS virus in France during the 1980s illustrates the magnitude of the risk. When xenotransfusions are considered in humans, the risk of the recombination of DNA sequences from the porcine endogenous retroviruses (PERVs) constitutes one of the principal ethical objections to clinical trials [36]. Cats may carry two retroviruses: the feline leukaemia virus (FeLV) and the feline immunodeficiency virus (FIV). Testing the donor for infection must be carried out if feline blood is to be used; the sensitivity of available in-house tests is very high [37].

Dogs do not carry FeLV or FIV retroviruses, and therefore do not need to be tested. By the same token, the dog is unable to transmit feline mycoplasma, an infection which is sometimes difficult to diagnose on blood smears, and for which the diagnostic gold standard (PCR) is not available for in-house use.

Due to of the risk of infection, feline blood donors should be chosen from cats that are adequately treated against fleas, and that are indoor-only [16,17,19,34]; these precautions need not be taken if the donor is a dog.

### 10.2. Under-Estimated Infection Risks

Regarding FeLV, detection of the p27 antigen allows avoiding donations from viraemic cats. There is nonetheless the possibility of a latent infection characterized by the absence of viraemia and the persistence of a virus within the bone marrow [38]. Cats suffering from a regressive infection develop a cellular and humoral immune response to FeLV and overcome viraemia within a few weeks; they test negative for the p27 antigen but remain carriers of provirus detectable by PCR for the rest of their lives [39]. The prevalence of cats which are positive for provirus and negative for the p27 antigen varies according to the population studied and may be as high as 10% [39,40]. These provirus-positive cats often possess a durable immunity against FeLV, but the reactivation of infection with the development of the illness associated with FeLV has been documented many times [38,41,42,43].

In an experimental study published in 2015, Nesina et al. transfused 10 specified pathogen-free (SPF) kittens with 10 mL of blood from cats that were p27-negative but were carriers of the FeLV provirus [44]. One cat developed a non-regenerative anaemia associated with FeLV-C, and four others developed a T-cell lymphoma, one with a secondary lymphoblastic leukaemia. A fatal illness was thus caused in 50% of the cats transfused. A total of 1 of the 3 donor cats, which were apparently healthy, died from T-cell lymphoma. This study demonstrates that proviral DNA in cats with a regressive infection can retain its ability to replicate for many years [42,43]. Appropriate PCR testing is recommended to identify provirus carriers in order to exclude them from blood donation [44].

Until 2020, the recommendations concerning transfusion in the cat [16,17] did not mention Nesina’s study published in 2015, but did warn of the risks of transmitting FeLV provirus. In a study published in 2018 examining the relevance of recommendations concerning feline transfusions, 1 cat of 31 enrolled tested positive for FeLV provirus but would not have been excluded from donation by current recommendations [45]. In 2021, a retrospective study lead by Mesa-Sanchez et al. in Spain and Portugal determined the prevalence of subclinical infectious agents in a large population of healthy, client-owned, indoor cats eligible to become blood donors [46]. A total of 8.1% (414/5105) had at least one subclinical infectious agent transmissible through blood transfusion; 1.5% were positive for FelV antigens, 2.9% for antibodies against FIV, 3.7% for haemoplasmas and 0.2% for Bartonella spp; among the 173 FeLV and FIV SNAP-negative cats tested for FelV provirus, 9 (5.2%) were positive.

The study by Nesina et al. calls into question the practice of allotransfusion in the cat; since the PCR test capable of detecting FeLV provirus is not available for in-house use, only cats previously tested by PCR which never go outside should be used as donors. No cat chosen extemporaneously should be used as a donor based only on its blood type and FeLV and FIV serological status (ELISA testing); biomolecular (PCR) screening for FeLV provirus is necessary.

Because the species barrier is crossed, and because most infectious agents are species-specific, the risk of transmitting an infectious agent from canine blood to a cat is very low; on the other hand, the risks of transmitting an infectious agent (and the illness associated with it) from cat to cat are significant.

## 11. Financial Constraints

Allotransfusions in cats are more costly than xenotransfusions with canine blood: sedation or anaesthesia, possible echocardiography, typing of recipient’s and donor’s blood (and possibly of several donors, if the first is not compatible), screening for FeLV and FIV infections, blood smears to look for *M. haemofelis*, PCR to detect carriers of FeLV provirus. These costs, on the order of EUR 200 (USD 220), are unnecessary if canine blood is used.

## 12. Inconvenience of the Procedure

The inconvenience of allotransfusion for the cat, in terms of time and work required, leads many individual veterinarians to give up transfusions, which are still absolutely critical. Respecting the species barrier deprives some cats of a lifesaving procedure. Many of the constraints associated with feline transfusion disappear if the donor is a dog (anaesthesia, blood typing of both donor and recipient, retrovirus detection, blood films).

## 13. Conclusions

Two arguments support the current position, which recommends the use of feline blood for transfusion to cats:

(1) Contrary to what has been long believed, the cat has natural antibodies against canine erythrocytes, and therefore there is a potential risk of severe transfusion reaction.
(2) The benefits of xenotransfusion are temporary: around 2–4 days, compared to up to 30 days with allotransfusion.

These arguments may be countered:

(1) The risk of transfusion reaction is only theoretical; no reaction of this type has been reported thus far in the 127 published cases.
(2) The duration of the effects of xenotransfusion is often enough to turn a corner, without need for a second transfusion [6,7,8,9,10,11,15,23]. If the anaemia reappears after 2–4 days, an allotransfusion may be given following conventional guidelines, outside of the setting of the presenting emergency, since a prior xenotransfusion does not increase the risks of subsequent allotransfusion.

The use of feline blood is based, above all, on the dogma of the prohibition of crossing the species barrier.

Numerous arguments support the use of canine blood for feline transfusions:

- Canine blood transfused to cats is often beneficial.
- Transfusion of canine blood to cats does not cause severe transfusion reactions; at least such a reaction has never been reported.
- Transfusion of canine blood to cats causes no clinical problems, even minor ones.
- Blood typing of donor and recipient is mandatory between cats; there is no such need if the donor is a dog.
- One of the feline blood types, type B, is relatively rare among cats, and finding a donor for such cats is sometimes difficult.
- Compatibility between cats is sometimes problematic [9].
- It is easier do draw blood from dogs; cats must sometimes be sedated or even anaesthetized.
- Canine blood may be collected in a closed system which is difficult at present in the cat.
- Canine blood may be collected by gravity while feline blood is aspirated which affects the quality of the product.
- The volumes withdrawn (about 60 mL) are negligible for a dog but sizeable for a cat.
- There is no risk of retrovirus or mycoplasma transmission from dogs to cats.
- A cat testing FeLV negative for antigen p27 may transmit the FeLV provirus, causing lymphoma in the recipient [44]; this risk disappears if the donor is a dog.
- Establishment of a feline blood bank is too great an endeavor for an organization which does not have a frequent need for feline transfusion.
- Emergencies do not always allow the determination of the recipient’s blood type [10,15].
- There are fewer constraints against transfusion if the donor is a dog.
- The cost of transfusion is much lower if the donor is a dog.
- Since there are fewer constraints, transfusion with canine blood is completed more quickly, which is significant in emergencies.
- Veterinarians may be led to forego a life-saving transfusion because of the unwieldiness of the procedure if the donor is a cat.

In the light of these arguments, when feline blood collected according to current guidelines is not available, in particular when the donor is not confirmed to be negative for the FeLV provirus, the authors consider it to be judicious to use canine blood for feline transfusion in emergency situations; this practice is preferable to inaction and to the inoculation of an infectious agent. Allotransfusion remains preferable in non-emergency situations as the treatment of chronic compensated anaemiae or if an appropriate donor (negative for FeLV provirus) is available. In 2–4 days after a xenotransfusion, if a clinical alteration and a significant decrease in haematocrit are observed, a transfusion with cat’s blood confirmed to be negative for FeLV provirus should be performed. Xenotransfusion should never be used twice.

## Data Availability

Not applicable.
